# Locomotion rhythm makes power and speed

**DOI:** 10.1038/s41598-023-41023-6

**Published:** 2023-08-28

**Authors:** A. Bejan, U. Gunes, H. Almahmoud

**Affiliations:** 1 Department of Mechanical Engineering and Materials Science, Duke University, Durham, NC 27708-0300 USA; 2 Department of Naval Architecture and Marine Engineering, Yildiz Technical University, Istanbul, 34349 Turkey; 3 Mechanical Engineering Department, King Fahd University of Petroleum and Minerals, 31261 Dhahran, Saudi Arabia

**Keywords:** Biophysics, Biotechnology, Evolution, Energy science and technology, Engineering, Physics

## Abstract

This article addresses two questions, why certain animals (frogs, breaststroke swimmers, hovering fliers, jellyfish) push rapidly against the surrounding fluid and then reach forward slowly, and whether this rhythm of propulsion is a manifestation of the universal phenomenon of design evolution in nature. Emphasis is on the distribution of time periods of locomotion in which, during the driving phase of cyclic movement (the motive stroke, phases 1 and 2, in alternating sequence with the dissipative stroke, phase 3), the work is generated (phase 1) and dissipated (phase 2). The relative lengths of the characteristic times t_1_ and t_2_ of the phases 1 and 2, are predicted. The relative duration of the proposed three phases of a cycle is the ‘rhythm’. The analysis is based on a model of how the effective cross-sections of the stroking body parts impact the surrounding medium, water, or air, and the total power required to account for the kinetic energy losses during phases 2 and 3, which are due to drag forces posed by the surrounding medium. The body configuration (limbs' cross-sections) determines the limbs' velocities that maximize mean power, and the times t_1_ and t_2_ within the motive stroke. Emphasis is placed on the freedom to change the evolving design. Freedom is represented in two ways: the number of degrees of freedom in changing the dimensions of the model and its deformation in time, and the effect that evolutionary changes have on the access that the body has to its available space. Freedom to change the locomotion design leads to greater power and speed.

## Introduction

Have you noticed that frogs and breaststroke swimmers push the water back quickly, and reach forward slowly? Did you also notice that after the push & reach motion their bodies ‘glide’ during a time interval comparable with the first two? Why is this happening? What is the connection between this rhythm and the evolution of animal design and its movement on earth?

In this article we address these questions in a fundamental way, based on theory. This work is predictive, as it relies on the physics law of design evolution in nature, the constructal law^1–19^. First, let us review the main terms and concepts that are employed in the article, which build bridges between physics, animal design, and engineering: locomotion, thermodynamics, fluid dynamics, design, model, scale, freedom, and evolution.

Locomotion (or transportation, for humans) is movement with purpose, to offer access to moving animals and people, from one location to another. Movement is against a medium—in water, on land, and in the air. Power drives movement, and all the power is dissipated as the moving body advances against the opposition posed by the medium. This is the thermodynamics of locomotion, in a nutshell.

Thermodynamics is broader than fluid dynamics^2^. Thermodynamics means heating (*therme*, Gr.) and power (*dynamis*, Gr.). Between the flows of heat and power there are macroscopic flow configurations (designs) that convert the heating into power and the movement driven by that power (called engines, animals, vehicles), or convert the power and movement into heating (called coolers, refrigerators, brakes, dissipators). The ‘heating’ originates from processes (combustion, metabolism) that consume measurable quantities known as food, fuel, exergy, free energy, caloric value, etc. The rate of consumption (e.g., metabolism) must not be confused with the power generated for causing movement (e.g., locomotion). Between the two there is the flow design that converts partially the first into the second.

Essential in thermodynamics and this article is the flow configuration—the design—its movement in space and time, and its evolution. Evolution means change after change in a direction discernible to the observer.

Regarding locomotion, fluid dynamics teaches the relation between the speed of the mover and the forces that the mover must overcome. Together, thermodynamics and fluid dynamics teach the relation between movement and the power required (and dissipated) to drive the movement. It is here that the human need is most evident. Because the power from fuel is not freely abundant, the motive behind most of the advances in fluid dynamics has been to decrease the power requirement when the needed speed and size of the mover are specified. The fluid dynamics of locomotion and transportation has generated a rich catalog of body configurations (designs), such as the hydrodynamic shape of fish, birds, hulls, keels, submerged wings, turbine blades and vanes, trucks, trains, automobiles, airplane fuselages, wings, and engine and wheel covers^1–5^.

The science of animal design benefits from physics, and transportation design finds inspiration in the animal realm^6–9^. These advances continue to attract questioning and revision. For example, the work of Hirt et al.^18^ was addressed in Günter et al.^20^ and Bejan et al.^6^.

Fluid dynamics developed because of two factors: the human need to have easier and faster transportation, as in the adoption of boating 100,000 years ago and domesticated animals 10,000 years ago^20–22^^,^ and the arrival of cheap power from fire (steam engines) and the industrial revolution. Ship propulsion and aviation are the main technologies that stimulated the fundamental advances that became fluid dynamics. Examples are airplanes during the twentieth century^23–36^, and wind power, boats with sails^37^ and maritime transport^6,38–41^.

## Background

**Figure 1:**
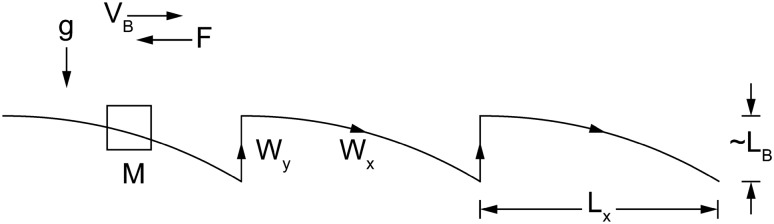
The two-step cycle of the theory in Refs.^1,4^.

The universal scaling laws of locomotion in all media came from the idea^1,4,5^ that for a body to move forward it must perform work and dissipate work (W_y_ and W_x_, Fig. 1). The body does work (W_y_) to lift its weight to a height that matches its linear dimension (length), and while falling forward it dissipates work (W_x_) to displace the medium in front of it. In swimming, the lifted weight is that of the parcel of water displaced by the body as it moves forward.

Unlike in the background reviewed below, the theory presented in this article is about the rhythm of motion during W_y_ alone, not during W_y_ + W_x_. It is about what happens during the motive stroke (t_y_) alone, which in Section “Rhythm” will be divided into two time intervals, doing work, t_1_, followed by dissipation, t_2_, in other words t_y_ = t_1_ + t_2_.

In the original theory^1,4,5^ the moving body has one length, mass M, or weight Mg. The time interval of ‘motive’ time, t_y_, must match the time interval of work dissipation during falling forward, t_x_. The single time interval (t_y_, or t_x_) is dictated by the body weight (Mg), namely (M/ρ)^1/6^g^–1/2^, or (L_B_/g)^1/2^, where ρ is the body density and L_B_ is the single body length, L_B_ ~ (M/ρ)^1/3^. Conversely, the frequency of the lift and fall rhythm must be lower in bigger bodies. This is in accord with animal locomotion and competitive athletics^4,42^.

**Figure 2:**
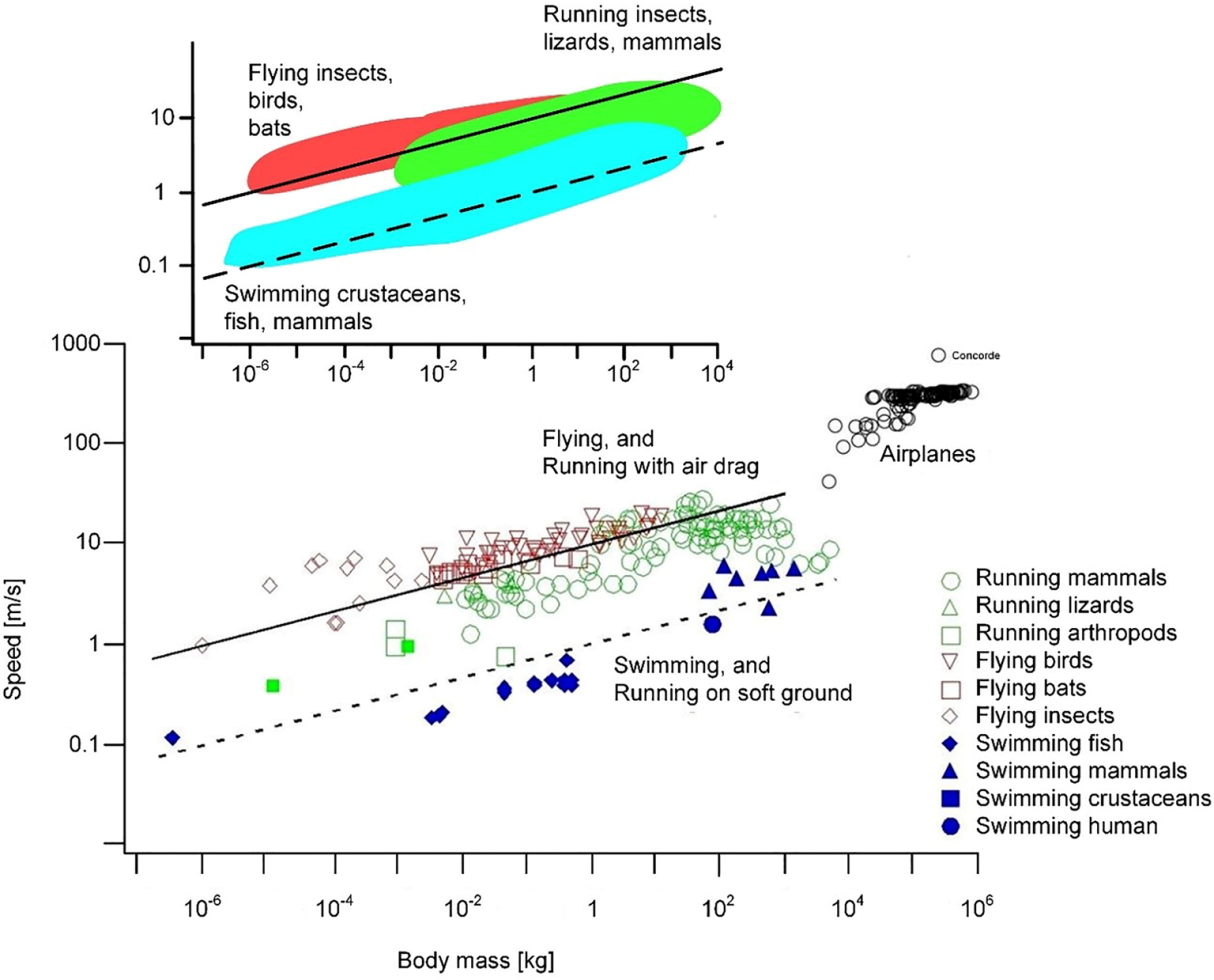
The universal scaling of steady locomotion in all media on earth (after Refs.^4–6^).

The freedom in the design is in the speed of locomotion, V, which is free to vary while decreasing the total effort (W_y_ + W_x_) made to cover the distance L_x_. The resulting speed is proportional to M^1/6^ (ρ/ρ_m_)^1/3^, where M is the body mass and ρ and ρ_m_ are the body and medium densities. The predicted (theoretical) lines are drawn in Fig. 2 for fliers and swimmers.

In sum, bigger movers should be faster (Fig. 2). Fliers should be approximately ten times faster than swimmers of the same size, because the factor (ρ/ρ_m_)^1/3^ in water is 1, and in air is 10. The frequency of the lift & fall cycle (f ~ V/L_x_) must be proportional to M^–1/6^, therefore the bigger movers should move their limbs less frequently. The work per unit travel must scale as (ρ_m_/ρ)^1/3^ Mg. Consequently, flying is approximately ten times more economical than swimming, because (ρ_m_/ρ)^1/3^ in air is 1/10, and in water is 1.

The factor (ρ_m_/ρ)^1/3^ is the physics of why bio designs spread from water to land and later in air. The ‘food chain’ is a consequence of the same physics. The bigger animal is faster and catches the smaller animal. The flier is faster and preys on the slower movers of the same size in water. The present article is not about legged locomotion, although the data covering terrestrial locomotion are included comparatively in the original theory^4,5^. For legged locomotion, the reader is directed to Günther et al.^20^.

These predictions unite the voluminous and highly diverse body of observations. Figure 2 shows the latest compilation over the body size range 10^–6^–10^8^ kg. The two straight lines are the theoretical scaling laws for fliers and swimmers. The spacing between the lines (fliers vs. swimmers) is the theoretical factor (ρ_m_/ρ)^1/3^ = 10, which unites locomotion in water with locomotion in air. The deviation of airplanes from the line is due to flying higher, because the air density for airplanes is lower than for animal fliers. The density difference was taken into account in the upper graph, which is the most recent^7^.

Figure 2 is instructive for an additional reason: it puts on display the physical meaning of the concept of ‘scale’. The segments on the abscissa and the ordinate cover many *different* orders of magnitude of their respective measurements. Coming from the Latin *scalae*, the word scale means flight of stairs, staircase, ladder, or a sequence of steps. The steps are marked as segments on rulers and tape measures used historically in design and manufacturing, from tailor shops to construction sites. An old slide rule is segmented in centimeters, therefore, it is a ‘centimeter scale’ to be used for measuring centimeter-scale bodies. The tape measure used in landscaping and construction is marked in meters, therefore, it is a meter-scale. The two scales are physically as different as the objects measured with them. That is why Fig. 2 is a multi-scale collection of measurements of animals with locomotion.

**Figure 3:**
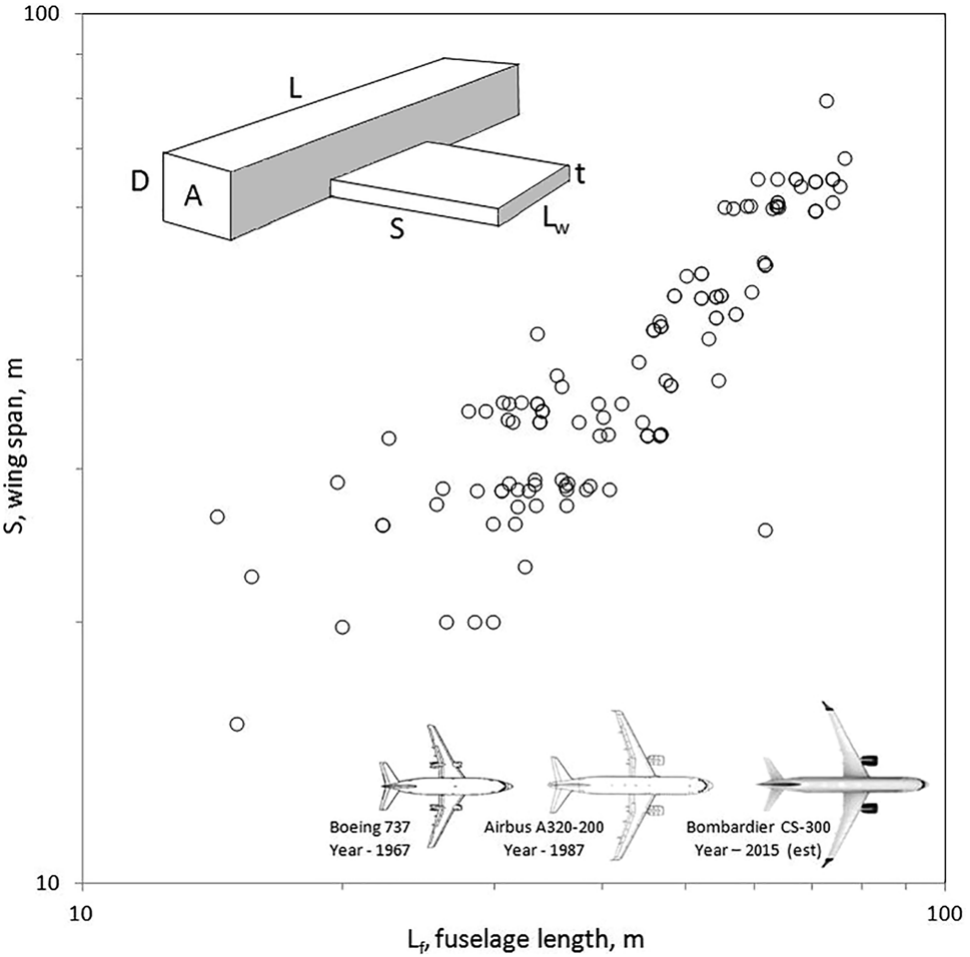
The proportionality between wingspan and body length is predictable^19^.

**Figure 4:**
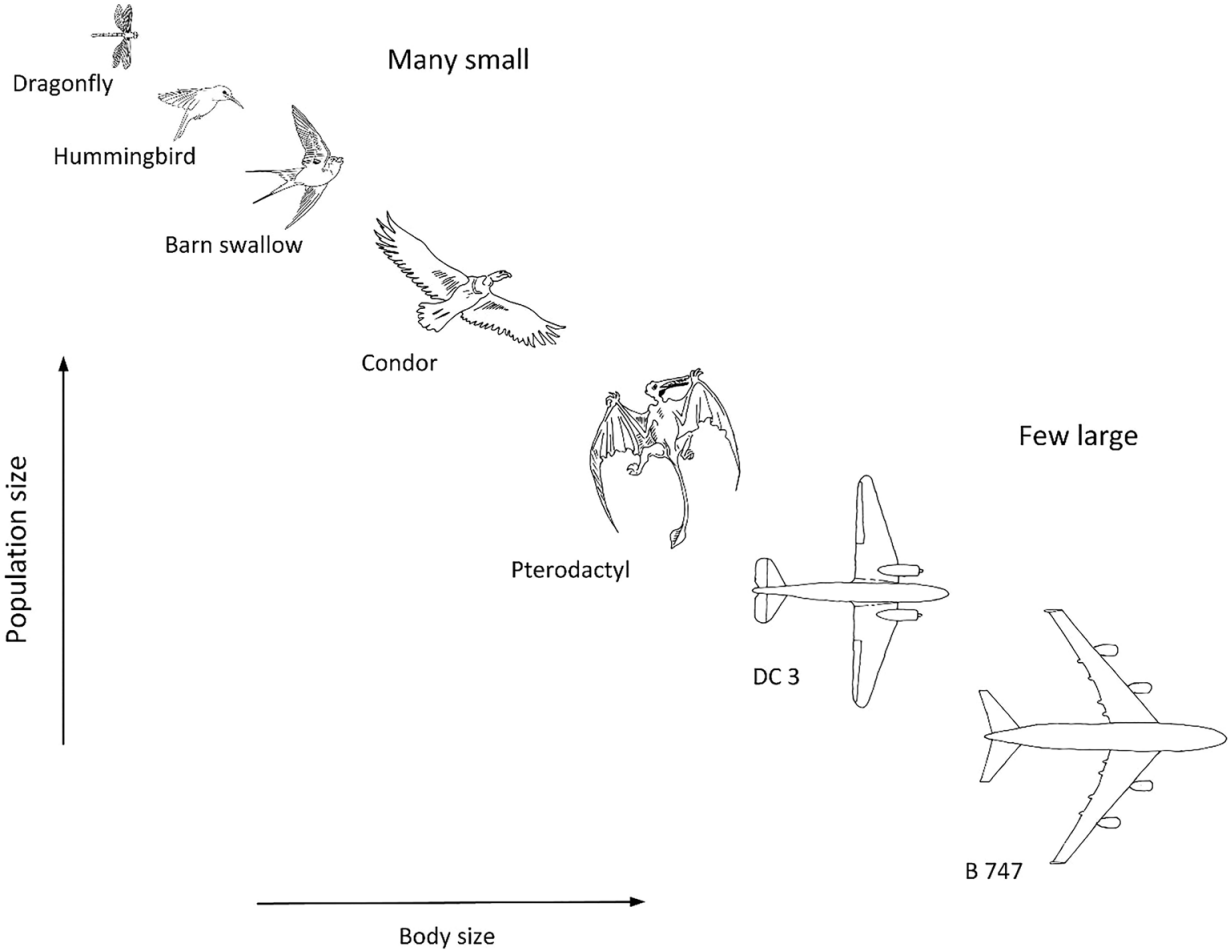
Animals and vehicles sweep the surface of the earth organized in hierarchies of body size and body numbers^22^.

The observed animal body is an object with many features, one large, some small, and many much smaller. If distinguishable, these features are different *length scales*. The large scale (say, L) is related to the body mass of the animal (M), therefore, if M is measured then the large length scale is known, L ~ (M/ρ)^1/3^, where ρ is the body density. For a person L is of order 1 m; smaller features (palms and feet) are of order 10 cm; many much smaller features (fingers, toes, feathers, hairs) are of order 1 cm and 1 mm. These three examples represent different length scales, cf., Figs. 3 and 4.

The body is not the object of analysis in science: it cannot be because it is too complicated to describe, manipulate, and argue about. The object of analysis is a simple facsimile of the observed object, which is called *model*. Models are diverse, few simple and many complicated, or few single-scale and many multi-scale. The simplest model of the animal body (L_B_, or M) is the ‘one-length’ model used in Fig. 1 and the construction of Fig. 2. Adding more features to the one-scale model makes the model more realistic and multi-scale. The first reviewer of the original manuscript of this article pointed out that the added features are all dictated by the body size (M), because on a small animal (e.g., frog) all the features are small. Well, they are all ‘small’ from the viewpoint of the observer, who is certain that a frog is smaller than a man. Yet, as explained in the preceding paragraphs, the smaller features are different length scales.

To avoid confusion, we used the advice provided by the reviewer and called the smaller features ‘lengths’, not length scales. In the model with multiple features, the smaller scales are accounted for in terms of *aspect ratios*. For example, the characteristic palm length divided by the body length is one aspect ratio. If in the ensuring analysis the aspect ratio is free to vary then that ratio is one *degree of freedom* in the design and performance based on that model. Degrees of freedom are also the ratios of time intervals in the rhythmic movement of the animal.

Advances continue to be made by endowing the design of the moving body with more degrees of freedom. If the model of the bird or airplane is endowed with several lengths (Fig. 3) instead of the single length associated with the body size M [namely L_B_ ~ (M/ρ)^1/3^], then, as we predicted analytically in Ref. 19 based on the constructal law, it is possible to determine the four shapes that account for the flying architecture: fuselage cross section A, fuselage profile L/D, wing cross section t/L_w_, and wing span divided by fuselage length, S/L. Figure 3 shows that the S/L proportionality predicted from theory is in accord with the evolution of commercial jet aircraft^19^.

The predicted architecture holds equally for animal flight, which is why airplanes have evolved to look like birds^43^. They evolved with freedom to acquire greater access as they move through the medium. They did not emerge from ‘mimicking’ bird design. The evolution of civil and military helicopters was predicted the same way as for airplanes, by evolving the multi-length architecture with freedom and consistently toward greater access, in accord with the constructal law. For example, the rotor diameter should match the body length scale of the helicopter^44^. Boats with sails, big or small, should look the same: the height of the sails should match the length of the hull and the mast^37^.

Movers on earth come in all sizes because they must scan the surface of the earth completely: they achieve this with hierarchies of body sizes and body numbers. Airplanes, birds, and ships should be organized as hierarchical traffic routes populated by many small and few large movers^22,45^, as is suggested qualitatively in Fig. 4. The biggest moves on earth—the river basins—share the same hierarchy, and for the same reason. Water and air currents evolve into hierarchies to have access on the globe, and their organization is predictable^22^. Extensive literature reviews are in Refs.^4,5,15^.

## Rhythm

The following theory is to regard locomotion from a ‘more liberated’ viewpoint. The idea is to endow the motive stroke with an additional degree of freedom: intermittency. The work motion is ‘push and reach’. The added freedom is the rhythm in progress forward. Rhythm is the duration of ‘push’ relative to ‘reach’ during the motive stroke (t_y_, Fig. 1).

**Figure 5:**
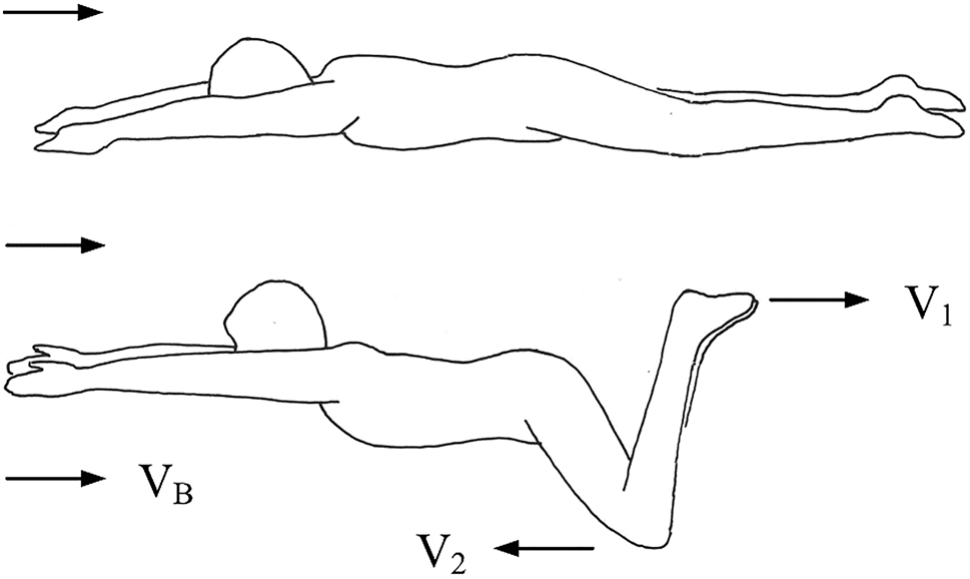
Breaststroke: the frog style of swimming is symmetric in space and periodic in time.

The analysis is presented in terms familiar to the reader: frog-like swimming (Fig. 5). The rhythm can be adjusted freely so that the time-averaged swim is faster per unit of power spent, or more economical (with reduced power) when the speed is specified.

The motion of the swimming frog is symmetric in space and periodic in time. The legs and the front members kick back symmetrically. The legs complete a cycle, the front members do the same, and together they complete the motive stroke of pushing forward (t_y_, Fig. 1).

The new feature in the present article is that the motive stroke is composed of two unequal time steps, t_1_ and t_2_. The back kick (t_1_) is shorter than the forward reach (t_2_). Why is this evolutionary design present in amphibians and in humans (breaststroke, Fig. 5)?

Speed is one objective, because a greater speed is essential for life. Amphibians swim away from danger, and they are not the only ones. Soldiers and freedom lovers swim across the river to save themselves. Athletes swim against the clock to win medals, fame, and opportunities for a better life after their competitive sports careers. The other objective is economy of effort, which is documented in many studies in zoology and biophysics^8–17^.

Size makes speed (Fig. 2) and gives the impression that the speed question has been answered. True, bigger bodies swim faster in the animal realm, where evolution (the freedom to change the design) had a very long time to run its course. It is not true in the swimming pool, where speed records do not come from the big bodies of obese swimmers. Speed records come from two distinct features of design, the body (size, shape, svelteness) and the rhythm in the morphing of the body shape.

Consider the simplest model that captures the main features of the moving design (Fig. 5). The body of the swimmer is represented by its mass M (kg), the single scale D (m) and the time-averaged forward speed V_B_ (m/s). That V_B_ is related to M is not the issue here: the challenge is to maximize V_B_ by varying freely the rhythm in a more realistic description of the body architecture.

As noted already, the two steps of the motive stroke (frog, breaststroke) are represented by two time intervals, t_1_ (back kick) and t_2_ (forward reach). In the frame of reference attached to the moving body, the back kick is executed with the speed V_1_ to a distance (the reach) of size L, which is comparable with the body dimension D, therefore t_1_ ~ L/V_1_. The body pushes against the water with a blunt member (leg, arm) with frontal cross section D^2^, which is fast so that the Reynolds number (based on D and V_1_) exceeds 10^2^, and consequently the drag coefficient C_D_ is a constant of order 1. In the following analysis we neglect factors of order 1 in accord with the rules of scale analysis^46^.

The same model captures the main features of the second step. In the frame of reference attached to the body, the forward reach is to a distance L of order D with the speed V_2_ during the time interval t_2_ ~ L/V_2_. The cross section of the blunt member (leg, arm) is D^2^, and the drag coefficient is constant and of order 1.

During t_1_ the legs and arms push back against the water and propel the body forward. The force felt by the body is

1 $$F_{1} \,\,\sim \,\,C_{D} \,D^{2} \,\frac{1}{2}\rho (V_{1} \,\, - \,\,V_{B} )^{\,2}$$

where ρ is the water density and C_D_ ~ 1, which in this analysis is neglected along with the 1/2 factor. The speed V_1_ refers to the movement of the arms and lengths in the frame of reference of the swimmer. If the length of the swing (arm, leg) is L,

2 $$V_{1} \,\,\sim \,\,\frac{L}{{t_{1} \,\,}}$$

the work done by the moving members is

3 $${\text{W}}_{{1}} \sim {\text{ F}}_{{1}} {\text{L}}$$

During t_2_ the legs and arms moved forward to the distance L. Their speed in the frame attached to the swimmer is

4 $$V_{2} \,\,\sim \,\,\frac{L}{{t_{2} \,\,}}$$

The relative speed between the moving members and the water is V_2_ + V_B_, therefore the drag force on the members is

5 $$F_{2} \,\,\sim \,\,C_{D} \,D^{2} \,\frac{1}{2}\rho (V_{2} \,\, + \,\,V_{B} )^{\,2}$$

The work done by the swimmer during t_2_ has the effect of a ‘brake’, and acts against propulsion:

6 $${\text{W}}_{{2}} \sim {\text{ F}}_{{2}} {\text{L}}$$

The periodic motion has several degrees of freedom that will become clear soon. Adjusting the variable features against each other is the key. In the present analysis the objective is a greater speed V_B_, which means greater net power $(\dot{W})$ spent on moving forward,

7 $$\dot{W}\,\,\sim \,\,\frac{{W_{1} \,\, - \,\,W_{2} }}{{t_{1} \,\, + \,\,t_{2} }}$$

or

8 $$\dot{W}\,\,\sim \,\,F_{B} V_{B}$$

where F_B_ is the averaged drag force on the body moving at speed V_B_:

9 $$F_{B} \,\,\sim \,\,C_{D} \,D^{2} \,\frac{1}{2}\rho V_{B}^{2}$$

From Eqs. (7)–(9) we conclude that $\dot{W}$ is proportional to $V_{B}^{3}$, which means that in the remainder of this analysis we focus on maximizing the function f defined below:

10 $$\frac{{\dot{W}}}{{\frac{1}{2}\rho D^{2} }}\,\, = \,\,f(V_{1} ,\,\,V{}_{2},\,\,V_{B} )\,\, = \,\,\frac{{(V_{1} \,\, - \,\,V_{B} )^{\,2} \,\, - \,\,(V_{2} \,\, + \,\,V_{B} )^{\,2} }}{{\frac{1}{{V_{1} }}\,\, + \,\,\frac{1}{{V_{2} }}}}$$

First, we consider V_1_ and V_B_ fixed, and vary V_2_ by noting that the function f is equal to $V_{1}^{3} \tilde{f}$, where $\tilde{f}$ is the dimensionless function

11 $$\tilde{f}(x)\,\, = \,\,\frac{{(1\,\, - \,\,\beta )^{\,2} \,\, - \,\,(x\,\, + \,\,\beta )^{\,2} }}{{1\,\, + \,\,\frac{1}{x}}}\,\, = \,\,x(1 - x - 2\beta )$$

where

12 $$x\,\, = \,\,\frac{{V_{2} }}{{V_{1} }},\,\,\,\,\,\,\,\,\,\,\,\,\,\,\beta \,\, = \,\,\frac{{V_{B} }}{{V_{1} }}$$

$\tilde{f}(x)$ is maximum at $x\,\, = \,\,\frac{1}{2}\,\, - \,\,\beta$, which means that V_2_ must be smaller than half of V_1_:

13 $$V_{2} \,\, = \,\,\frac{{V_{1} }}{2}\,\, - \,\,V_{B}$$

The maximum power forward that corresponds to Eq. (13) assumes the form

14 $$\dot{W}_{\max } \,\,\sim \,\,\frac{1}{2}D^{2} \rho V_{1}^{3} \left( {\frac{1}{2}\,\, - \,\,\frac{{V_{B} }}{{V_{1} }}} \right)^{\,2}$$

which, in view of $x\,\, = \,\,\frac{{V_{2} }}{{V_{1} }},\,\,\beta \,\, = \,\,\frac{{V_{B} }}{{V_{1} }}$ and Eqs. (8) and (9), leads to one equation for V_B_/V_1_:

15 $$\left( {\frac{1}{2}\,\, - \,\,\frac{{V_{B} }}{{V_{1} }}} \right)^{\,2} \,\, = \,\,\left( {\frac{{V_{B} }}{{V_{1} }}} \right)^{\,3} \,$$

Although the exact solution is V_B_/V_1_ = 0.3194, for simplicity we will use

16 $$V_{1} \,\, \cong \,\,3V_{B}$$

Next, from Eqs. (13) and (16) we obtain

17 $$V_{2} \,\, \cong \,\,\frac{1}{2}\,V_{B} \,\, \cong \,\,\frac{1}{6}\,V_{1}$$

18 $$\dot{W}_{\max } \,\, \cong \,\,\frac{3}{8}\,\rho D^{2} V_{B}^{3}$$

In conclusion, we can predict that for greater speed in swimming the reaching motion should be slower than the kicking motion. This agrees with observations of swimmers, as we will show in Sections “Frog swimming” and “Breaststroke”.

The total power $\dot{W}_{tot}$ spent by the swimmer is greater than $\dot{W}_{\max }$ because the morphing body performs work in both directions, pushing back (W_1_) and reaching forward (W_2_). The scale of $\dot{W}_{tot}$ is derived by replacing Eq. (7) with $\dot{W}_{tot} \,\,\sim \,\,(W_{1} \,\, + \,\,W_{2} )/(t_{1} \,\, + \,\,t_{2} ),$ and then using the results of the $\dot{W}_{\max }$ analysis above, namely Eqs. (16) and (17). The result is

18' $$\dot{W}_{tot} \,\, \cong \,\,\frac{51}{{28}}\,\rho D^{2} V_{B}^{3}$$

Dividing Eqs. (18) and (18'), we find that the net power spent on moving forward is roughly 1/4 of the power spent by the swimmer.

Finally, the scale $\dot{W}\,\, \cong \,\,D^{2} \rho V_{B}^{3}$ represents both efforts, Eqs. (18) and (18'). Inserting in this $\dot{W}$ scale the V_B_ scale predicted by minimizing effort per unit travel^3,4^, namely V_B_ ~ (gD)^1/2^, we find that the work spent per unit travel is of order Mg (or ρD^3^g), which is in accord with the earlier theory of locomotion^1,4,5^ (cf., Section “Background”).

## Two lengths

**Figure 6:**
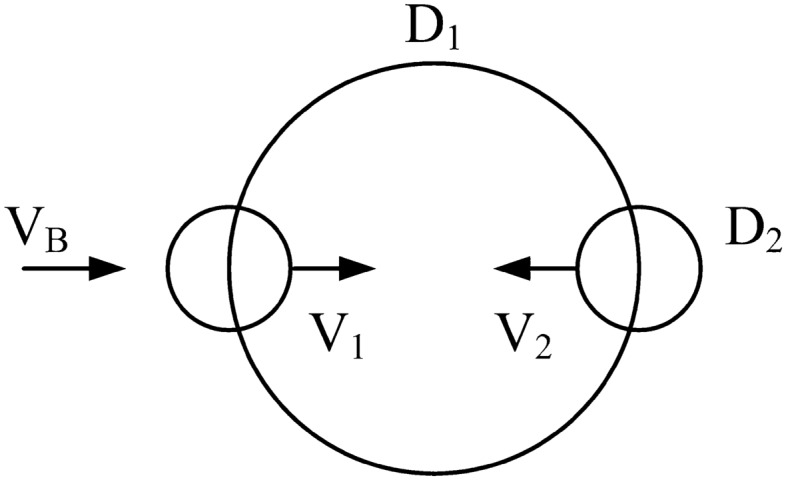
Body with two lengths scales: the body scale D_1_, and the moving organs D_2_.

The one-length model (D) of the body with rhythmic movement in water (Fig. 5) can be extended to more accurate descriptions. The simplest step in this direction is in Fig. 6. There are at least two lengths that capture the body construction, the frontal cross section of limbs that push against the water, $D_{2}^{2}$, and the frontal cross section of the torso (shoulders, head, etc.), $D_{1}^{2}$. The length of the movement of the limbs (during kicking and reaching forward) continues to be represented by the body scale D_1_.

In the two-length model the new degree of freedom is the relative size of the moving parts, namely the ratio ε = D_2_/D_1_, where ε < 1. Interesting and potentially useful in the conceptual design of artificial locomotion is to discover what aspect ratio D_2_/D_1_ characterizes a better design, the faster swimmer, and the more economical aquatic robot.

The analysis follows in the same steps as in Eqs. (1)–(18) while keeping in mind the difference between the two lengths pictured in Fig. 6, namely D_1_ and D_2_ (or εD_1_). Equations (1) and (5) are replaced by

19 $$F_{1} \,\,\sim \,\,C_{D} \,\varepsilon^{2} D_{1}^{2} \,\frac{1}{2}\rho (V_{1} \,\, - \,\,V_{B} )^{\,2}$$

20 $$F_{2} \,\,\sim \,\,C_{D} \,\varepsilon^{2} D_{1}^{2} \,\frac{1}{2}\rho (V_{2} \,\, + \,\,V_{B} )^{\,2}$$

Equations (10), (14) and (15) are replaced by

21 $$\frac{{\dot{W}}}{{\frac{1}{2}\rho \varepsilon^{2} D_{1}^{2} }}\,\, = \,\,f(V_{1} ,\,\,V{}_{2},\,\,V_{B} )\,\, = \,\,\frac{{(V_{1} \,\, - \,\,V_{B} )^{\,2} \,\, - \,\,(V_{2} \,\, + \,\,V_{B} )^{\,2} }}{{\frac{1}{{V_{1} }}\,\, + \,\,\frac{1}{{V_{2} }}}}$$

22 $$\dot{W}_{\max } \,\,\sim \,\,\frac{1}{2}\varepsilon^{2} D_{1}^{2} \rho V_{1}^{3} \left( {\frac{1}{2}\,\, - \,\,\frac{{V_{B} }}{{V_{1} }}} \right)^{\,2}$$

23 $$\varepsilon^{2} \left( {\frac{1}{2}\,\, - \,\,\frac{{V_{B} }}{{V_{1} }}} \right)^{\,2} \,\, = \,\,\left( {\frac{{V_{B} }}{{V_{1} }}} \right)^{\,3} \,$$

**Figure 7:**
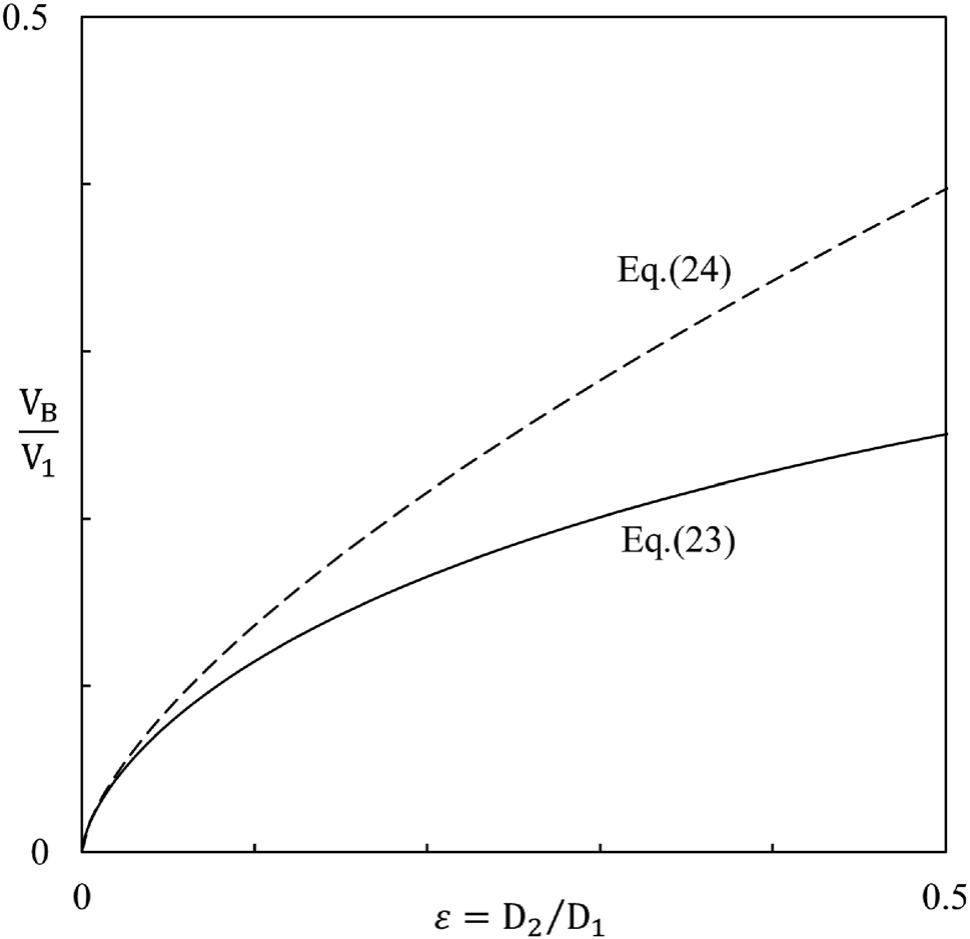
The relation between V_B_/V_1_ and D_2_/D_1_.

Equation (23) is plotted in Fig. 7. When ε approaches 1 the solution reproduces Eqs. (16)–(18') of the single-length model. More relevant is the opposite limit, ε << 1, because in swimmers the ratio D_2_/D_1_ is of order 1/10. According to Eq. (23), in the small-ε limit V_B_/V_1_ becomes negligible relative to 1/2 (inside the parentheses), and Eq. (23) reduces to, in accord with Fig. 7^,^

24 $$\frac{{V_{B} }}{{V_{1} }}\,\,\sim \,\,\left( {\frac{\varepsilon }{2}} \right)^{2/3} \,\, = \,\,\left( {\frac{{D{}_{2}}}{{2D_{1} }}} \right)^{2/3}$$

In place of Eqs. (17) – (18') we obtain

25 $$V_{2} \,\,\sim \,\,V_{1} \,\left[ {\,\frac{1}{2}\,\, - \,\,\left( {\frac{\varepsilon }{2}} \right)^{2/3} } \right]$$

26 $$\dot{W}_{\max } \,\,\sim \,\,\left( {\frac{1}{2}\,\, - \,\,2^{ - 1/3} \varepsilon^{4/3} \,\, + \,\,...} \right)\,\rho D_{1}^{2} V_{B}^{3}$$

26' $$\dot{W}_{tot} \,\,\sim \,\,\frac{5}{6}\,\,\left[ {1\,\, - \,\,\frac{4}{15}\,\,\left( {\frac{\varepsilon }{2}} \right)^{2/3} \,\, + \,\,...} \right]\,\rho D_{1}^{2} V_{B}^{3}$$

where “ + …” indicates higher order terms in ε, which are neglected. In the limit D_2_ <  < D_1_ the ratio $\dot{W}_{\max } /\dot{W}_{tot}$ approaches 3/5, indicating a higher fraction of power allocated to forward propulsion than in the result based on the one-length model [read under Eq. (18').

The main results of this section are compared in Table 1 against the corresponding results based on the one-length model (Section “Rhythm”). According to both models, $\dot{W}_{\max }$ and $\dot{W}_{tot}$ have the same scale when based on the larger dimension (D, or D_1_). The results for $\dot{W}_{\max }$ are close, indicating that the simpler model captures sufficiently well the value of the rhythm-maximized power for forward motion.

**Table 1:** The effect of the number of model lengths on the maximized external power for swimming forward, relative to the total internal power spent by the morphing body.

One length, D	Two lengths, D_1_ >> D_2_
$$\dot{W}_{\max } \,\, \cong \,\,\,\frac{3}{8}\,\,\rho D^{2} V_{B}^{3}$$	$$\underset{\raise0.3em\hbox{$\smash{\scriptscriptstyle\thicksim}$}}{ > } \,\,\,\frac{1}{2}\,\,\rho D_{1}^{2} V_{B}^{3}$$
$$\dot{W}_{tot} \,\, \cong \,\,\,\frac{51}{{28}}\,\,\rho D^{2} V_{B}^{3}$$	$$\underset{\raise0.3em\hbox{$\smash{\scriptscriptstyle\thicksim}$}}{ > } \,\,\,\frac{5}{6}\,\,\rho D_{1}^{2} V_{B}^{3}$$

The results for $\dot{W}_{tot}$ show that the total power responsible for the same $\dot{W}_{\max }$ is sizably smaller (by 46 percent in the limit ε << 1) in the two-length model, relative to the one-length model. This is an important finding because the change from the one-length model to the two-length model represents the direction toward more degrees of freedom in morphing the flow architecture of the swimming body. In the direction toward more freedom to change, the performance of the multi-length design is the recipient of rewards from freedom. Such rewards are a common occurrence in the evolution and performance of flow architectures with freedom to morph^22,47,48^. We return to this conclusion in Section “Discussion and conclusions”.

## Frog swimming

The frog derives most of its swimming power from the hind legs. The frog ‘motor’ is at the back. The swimming cycle is composed of three time intervals, in order:

- *Reach* The knees bend and move into the body of the frog. This time interval is t_2_ in the terminology of Section “Rhythm”.
- *Kick* The legs push back and become straight. This interval is t_1_ in the terminology of Section “Rhythm”.
- *Glide* The whole body shapes itself hydrodynamically (elongated, without abrupt changes in its cross section perpendicular to the swimming direction), and moves as if rigid while drag dissipates the kinetic energy acquired during t_1_. The time to glide is called t_3_.

**Figure 8:**
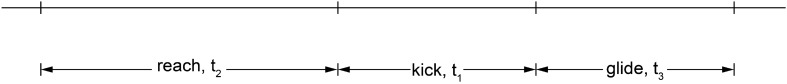
Three time intervals in the frog swimming cycle: reach, kick, glide.

The three time intervals are available for measuring in several videos of swimming frogs. The measurements reported in Fig. 8 are from watching a slow-motion version of the video available at^49^. The measured rhythm is such that if the total cycle (t_2_ + t_1_ + t_3_) has a duration of 7 time units, then $t_{2} \,\, \simeq \,\,3$ units, $t_{1} \,\, \simeq \,\,2$ units, and $t_{3} \,\, \simeq \,\,2$ units. Important are the ratios that these intervals reveal:

- t_1_ and t_2_ have the same scale, and $t_{1} /t_{2} \,\, \simeq \,\,2/3\,\, < \,\,1$, or $V_{2} /V_{1} \,\, < \,\,1$. This ratio is in accord with the one-length and two-length analyses of Sections “Rhythm” and “Two lengths”.
- The motor time interval is t_2_ + t_1_. In the earlier scaling of locomotion^3,4^ the gliding time t_3_ is the time of ‘falling forward’ against drag (t_x_ in Fig. 1). The earlier theory predicted that the motor time (t_y_) and the gliding time should have the same time scale. This is confirmed by the measurements plotted in Fig. 8, where t_3_/(t_2_ + t_1_) is of order 1.

## Breaststroke

The breaststroke swimmer benefits from the power furnished by two ‘motors’, one in front (the arms), and the other at the back (the legs). Each motor goes through the reach & push cycle described in Fig. 8 for the single motor of the frog. The two cycles of the arms and the legs are followed by an interval of gliding to dissipate the kinetic energy acquired from the motors.

**Figure 9:**
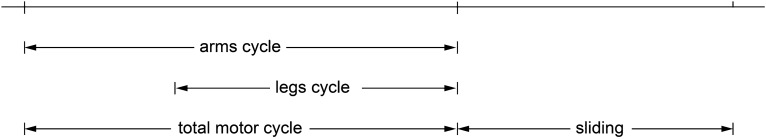
Three intervals in the breaststroke cycle: arms cycle, legs cycle, and sliding.

The time intervals of reach, push and glide are available on many videos of swimming lessons for amateurs and athletes. The measurements summarized in Fig. 9 came from watching the video available at^50^. The units of time on the horizontal scale are not important, as they depend on speed selected for the slow-motion examination of the video. Important are the relative sizes of the cycles and their internal reach & push intervals.

One complete cycle consists of one ‘arms cycle’, followed smoothly by one ‘legs cycle’ and then by one glide interval. The complete cycle (motor + sliding) repeats itself nearly identically as one watches the video. For example, a typical complete cycle began at time 0 and exhibited these changes:

0: arms begin to push back;
7: arms stop pushing back and begin to reach forward;
  legs begin to bend, heels approaching buttocks;
13: legs start to kick back;
16: legs are completely extended;
  sliding begins
17: arms stop reaching forward;
26: sliding ends, arms begin to push back, and so on.

Note that the arms cycle and the legs cycle overlap, and that together they constitute the motive interval that started at time 0 and ended at 16. This sequence of changes is illustrated in Fig. 9, where we plotted the averages of observations of four nearly identical cycles. The dissipative interval started at time 16 and ended at 26.

Three conclusions follow. First, the time ratio for push/reach during the arms cycle, t_1_/t_2_, is less than 1, in accord with the theory and with the measurements of rhythm in frog swimming. Second, the same conclusion holds for the push/reach time ratio of the cycle executed by the legs. Third, the total motor interval is of the same order as the sliding interval, in accord with the earlier theory of locomotion (Fig. 1, Section “Background”).

## Hovering

**Figure 10:**
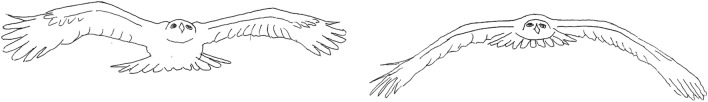
Two time intervals in flapping flight: closing and raising the wings above the torso, and spreading the wings and pushing the air fast downward.

Flapping flight (Fig. 10) is an opportunity to extend the theory to a science and technology that is even wider than its aquatic counterpart. In hovering, the body maintains its altitude in two steps, spreading the wings and pushing the air fast downward, and closing the wings and raising them above the torso. The net work performed during one cycle is represented by W_y_ in Fig. 1. In hovering the flapping is happening in place, which means that L_x_ = 0 and W_x_ = 0.

The work done during the first step (t_1_, downward) must be greater than the work done while raising the wings (t_2_). The wing (its face area) and downward speed must be greater than during the return motion (upward). Unknown, and potentially useful for designs of hovering flight are the rhythm (t_1_/t_2_) and the effect of changing the frontal area when the wing changes direction.

**Figure 11:**
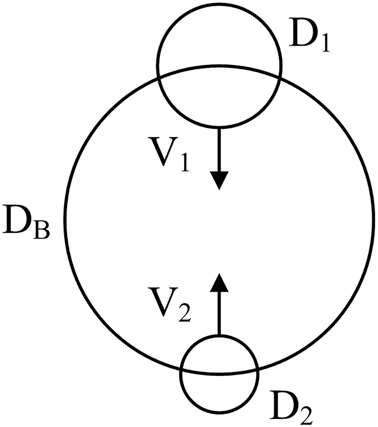
Model of hovering body with three length scales: D_B_, D_1_ and D_2_.

The simplest model is sketched in Fig. 11, where the frame of reference is attached to the body (D_B_). This model differs from that of Fig. 6 in three respects: it has three lengths not two, the motion is oriented vertically, and the length of the moving part changes from D_1_ (downward) to D_2_ (upward). The three lengths are (D_B_, D_1_, D_2_), where D_B_ is the length of the body as a whole $(M\,\,\sim \,\,\rho D_{B}^{3} )$, and M is the body mass.

Interesting in a fundamental sense is to predict the effect of changing sizes (D_1_, D_2_) on the overall performance of the moving body. During downward motion the frontal area of the wings (~ ${\text{D}}_{{1}}^{{2}}$) is greater than during return motion upward (~ ${\text{D}}_{{2}}^{{2}}$). The drag force, flap time, and work done by the wings while moving downward and upward are

27 $$F_{1\,} \,\sim \,\,D_{1}^{2} \frac{1}{2}\rho_{a} V_{1}^{2} ,\,\,\,\,\,\,\,\,\,\,\,\,t_{1} \,\,\sim \,\,\frac{{D_{B} }}{{V_{1} }},\,\,\,\,\,\,\,\,\,\,\,\,W_{1} \,\,\sim \,\,F_{1} D_{B}$$

28 $$F_{2\,} \,\sim \,\,D_{2}^{2} \frac{1}{2}\rho_{a} V_{2}^{2} ,\,\,\,\,\,\,\,\,\,\,\,\,t_{2} \,\,\sim \,\,\frac{{D_{B} }}{{V_{2} }},\,\,\,\,\,\,\,\,\,\,\,\,W_{2} \,\,\sim \,\,F_{2} D_{B}$$

The air density is ρ_a_, and the length of the vertical stroke is the body scale, D_B_. The total work done for keeping the body aloft during one cycle (t_1_ + t_2_) is

29 $$W\,\sim \,\,D_{B} \,D_{1}^{2} \,\frac{1}{2}\,\rho_{a} (V_{1}^{2} \,\, - \,\,\varepsilon^{2} \,V_{2}^{2} )$$

where $$\varepsilon \,\, = \,\,D_{2} /D_{1} \,\,\underset{\raise0.3em\hbox{$\smash{\scriptscriptstyle\thicksim}$}}{ < } \,\,1$$. The total power spent during one cycle is

30 $$\frac{W}{{t_{1} \,\, + \,\,t_{2} }}\,\sim \,\,D_{1}^{2} \,\frac{1}{2}\,\rho_{a} V_{1}^{3} \,\,\frac{{1\,\, - \,\,\varepsilon^{2} x}}{{1\,\, + \,\,\frac{1}{x}}}$$

where x = V_2_/V_1_. On the right side, the function of x reaches its maximum at the x value obtained by solving this implicit equation:

31 $$\varepsilon^{2} \, = \,\,\frac{{1\,\, - \,\,e^{ - 2x} }}{{2\,x^{3} }}$$

Equation (31) expresses x (or V_2_/V_1_) as a function of ε (or D_2_/D_1_). Here are three cases:

**Table:** 

ε, or D_2_/D_1_	1/2	3/4	1
x, or V_2_/V_1_	1.222	0.906	0.726
1 – ε^2^x^2^	0.627	0.538	0.473

The significance of the expression (1 − ε^2^x^2^), and how to calculate it, is explained next.

The maximum work available for maintaining the altitude of the body during one cycle is found from Eq. (29) by substituting in it the solution obtained for x(ε) by solving Eq. (31)^,^

32 $$W_{\max } \,\sim \,\,D_{B} D_{1}^{2} \,\frac{1}{2}\,\rho_{a} V_{1}^{2} \,(1\,\, - \,\,\varepsilon^{2} x^{2} )$$

$W_{\max }$ equals the work required to lift the body back to a height equal to the body length scale:

33 $$W_{lift} \,\sim \,\,\rho \,D_{B}^{3} \,\,g\,D_{B}$$

The body density ρ is significantly greater than the air density ρ_a_. Setting W_max_ ~ W_lift_, we obtain

34 $$\tilde{V}_{1} \,\sim \,\,\frac{1}{\eta }\left( {\frac{{\rho /\rho_{a} }}{{1\,\, - \,\,\varepsilon^{2} x^{2} }}} \right)^{\,1/2}$$

where η = D_1_/D_B_ $$\underset{\raise0.3em\hbox{$\smash{\scriptscriptstyle\thicksim}$}}{ < }$$ 1, and $$\tilde{V}_{1}$$ is the downward wing speed nondimensionalized relative to the Galileian speed of free fall to the level D_B_,

35 $$\tilde{V}_{1} \, = \,\,\frac{{V_{1} }}{{(2gD_{B} )^{1/2} }}$$

The effect of the aspect ratio D_2_/D_1_ (or ε) on W_max_ and $\tilde{V}_{1}$ is represented by the group $1\,\, - \,\,\varepsilon^{2} x^{2}$ reported in the above table. As D_2_/D_1_ increases, W_max_ decreases and $\tilde{V}_{1}$ increases. These variations are minor in the small range covered by D_2_/D_1_ for wings, meaning that important are the orders of magnitude and trends of these results.

One extension of the hovering analysis applies to aquatic movement. Consider the two-stroke body change employed by a jellyfish^51^. The extended canopy contracts as it ejects a part of its water volume. During the return stroke the canopy contracts and swells slowly to minimize the slowdown effect of the drag. In both strokes the tradeoff is between the time scales of pushing (t_1_) and reaching upward (t_2_).

At first sight, the jellyfish cycle looks like Fig. 11 and, certainly, the analytical treatments will be related. The difference is that the tendency to maximize the net work per cycle in the jellyfish comes from the need to move forward (or upward) as a neutrally buoyant body (the body of the jellyfish is 95% water). In flapping hovering (Fig. 11), the need is to stay aloft, i.e., to balance the body weight against gravity.

## Discussion and conclusions

We showed that when the rhythm is free to change, the moving body evolves toward economy of power and greater speed. The theory started with the idea that the rhythm is the unknown—it is not to be assumed as given. This point of view empowers us to fast-forward design ideas (images) and their evolution in the mind. In all of nature, flows are not happening in ‘given’ spaces and within rigid boundaries, or "boundary conditions". The flows carve and define their own spaces, configurations, and rhythms in time.

Configurations evolve because they have freedom to change. Every change in configuration (form, rhythm) opens the door to changes in the performance of the flow system. Every additional degree of freedom in the optimization of the movement is a step toward seeking economy of power and greater speed. We showed this quantitatively at the end of Section “Two lengths”, by comparing the one-length and two-lengths models. In competitive swimming, more degrees of freedom are available for changing the body configuration. One way is by properly sizing the spacings between the fingers of the open palms (cf. Ref.^5^, p. 108). More dimensions are available from varying and selecting (optimizing) the spacings between the toes.

Step by step, taking advantage of more degrees of freedom is accompanied by design phenomena that slow down the increase in performance and, in artificial objects, dampen the enthusiasm of the designer. Starting in the 1980s, our group has shown quantitatively and graphically that the world of imaginable designs is divided into two realms, *the possible* and *the impossible*^1,22,52–55^. Unlike the Carnot efficiency of power plants, which is an absolute (fixed) limit, the frontier between the possible designs and the impossible designs can move, slowly or abruptly, because of random events in nature, and sparks of ingenuity in the human minds.

In the wake of abrupt events evolution is reinvigorated, anything goes, and what works is kept. The performance of possible designs migrates toward the performance reached earlier by ‘champion’ designs that mark the existing frontier between the possible and the impossible. Migration is due to freedom to change and optimize each design. The design phenomenon that slows down the approach to the frontier is the physics of *diminishing returns*, cf., ch. 10 in Ref.^22^. On the same route, the design phenomenon of *economies of scale* gives birth to designs of *hierarchy*, arborescence, networks, and social organization, cf., ch. 2 in Ref.^22^.

Another reviewer objected that we “failed to evaluate (our) theoretical predictions in light of real data—i.e., measurements of cost transport in animals and machines across sizes (e.g., Tucker^56^). The reviewer’s objection is about the 2000–2006 theory^1,4,5^, not about the present article, because the theory of this article (rhythm with more than one degree of freedom) was tested here against measurements, in Figs. 8 and 9.

As we explained in Section “Background”, the theory of 2000–2006 invoked a single degree of freedom, and predicted a single result, which could be reported in three ways that are equivalent: speed, frequency, and work spent per unit of distance traveled. The theory of 2006^4,5^ was tested successfully against a large body of measurements from four independent groups of investigators. The test is summarized in ‘cloud’ form here, in Fig. 2.

One test validates the single result from a theory based on a body model with only one degree of freedom. In other words, the speed-mass test shown in detail in 2006 and here in Fig. 2 validated the alternate versions of the prediction, the frequency-mass prediction, and the work-mass prediction. The theory was not questioned and refuted by anyone. On the contrary, it was featured as an invited lecture at the 2004 workshop on animal design in Ascona^57^, and it was awarded the Benjamin Franklin Medal in 2018.

Independent confirmation of the theory^4^ is available throughout the animal design literature, as we found in the data projected on Fig. 2. While writing this discussion we came across two independent reports that validate the theory. When nondimensionalized as Strouhal number (St), the frequencies of wake undulations behind swimmers^58^ and fliers^59^ exhibit St values in the same range 1–10. The predicted frequencies that stand behind the theoretical lines in Fig. 2 agree with that universal Strouhal number range 1–10.

The Strouhal number is a dimensionless way of expressing the frequency of vortex shedding (or meandering) of turbulent (i.e., relatively inviscid) streams: jets, wakes, plumes. Its empirical discovery and relative universal value in the range 1–10 (independent of Re) was labeled St by its discoverer Vincenc Strouhal in 1878. It is a feature of large scale turbulence structure. The universal St range was predicted^60^ from pure theory in 1981, and reviewed in chapter 4 of Bejan’s 1982 thermodynamics book^52^.

All bodies moving in a medium (air, water) shed behind them vortices migrating with a particular frequency downstream as meandering wakes. The flows generated behind moving animals are recent examples in a body of St literature that validates through frequency measurements the universality of the St prediction made for turbulent flow in general^46,52,60^.

Although this confirmation should be enough, the theory^4^ revealed an additional part of the physics that continues to be overlooked in biology. The studies of wake frequencies behind swimmers^58^ are based on the classical view that gravity does not matter to the fish. The study of wakes behind fliers^59^ found that the Strouhal number for fliers is comparable with the Strouhal number for swimmers. Such a coincidence should have been questioned before, because obviously gravity does matter to the bird. Gravity is one of the two major forces in flight (the other is drag). The theory^4,5^ predicted this coincidence even before the question arose. We showed^1,4,5^ that the reason for the universality of the Strouhal number (or frequency) correlation is that gravity is as important in swimming as in flying.

**Figure 12:**
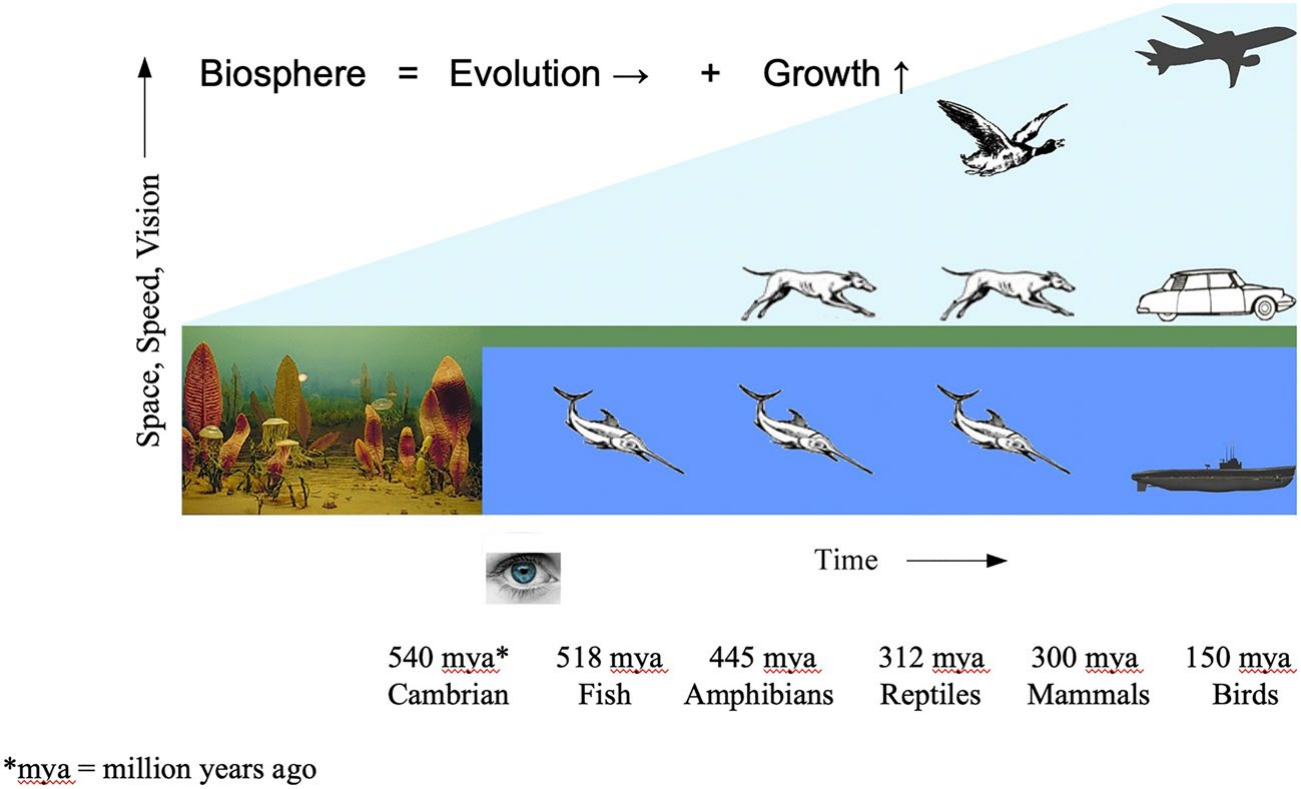
Timeline of the evolution and growth (spreading) of locomotion on earth.

Relevant to the reference made by the reviewer to Tucker^56^ is the tested prediction^4,5^ that the work/distance requirement for the swimmer is greater than for the runner and the flier (Section “Background”). This prediction is in accord with the history of the biosphere: the effort required for locomotion *decreased* chronologically in the sequence water-land-air. See Fig. 12 for the evolution and spreading of locomotion in the biosphere^61^: Big history contradicts Tucker^56^^,^ who claimed that the energy cost of locomotion decreases from running to swimming and, finally, to flying. Clearly, Tucker made a mistake, which was taken as truth by many including his Duke colleague Vogel^10^.

Our objective is not to criticize the work and legacy of our Duke colleagues. For them we have the greatest respect because of their contributions to the science of animal design^8–10^ and to the fame of our university. The disagreement stems from how the ‘cost’ of locomotion was evaluated. There are two ways to do this.

Our way, here and in the original theory^1,4,5^, was to focus on the thermodynamics (physics) of locomotion, the body and its actual movement against the ambient. The required work is due to a finely tuned cycle of two efforts, the work needed to lift the body, and the work needed to move forward by getting the environment out of the way. These two efforts are easy to rationalize, based on the simplest dynamics analysis. The derivation of W_y_ and W_x_ lies naked on the table^1,4^.

The other approach is older, and it is preferred in biology. It begins with the notion that life is due to the ‘free’ energy embodied in food, and that the power generated by metabolism drives locomotion and other functions inside the body. This approach is opaque because between the food intake and the movement of the body on earth there is an ‘engine’ that propels the body intermittently, not in sync with the food intake, which is also intermittent. An engine is a flow system with configuration (design). This, and the design and performance of the engine are not revealed. Coming from thermodynamics^2,52,53^ we know what is missing, starting with the concept of free energy (exergy, availability) that dates from Maxwell and Gibbs, not Schrödinger one hundred years later.

Tucker^56^ followed the second approach. He plotted on the ordinate the group P_i_/(WV), where W is the body weight (Mg in our article), V is the speed, and P_i_ is “the rate of energy release called *metabolic rate*”, which he called “the power input P_i_, a term more consistent with engineering usage”. No, it is not consistent with engineering, because both animal and vehicle do not move constantly while eating constantly. Furthermore, in a vehicle there is a complicated path of exergy destruction (irreversibility) from the combustion flame to the wheels of the vehicle. On the abscissa Tucker indicated the body mass, M. Based on such a plot Tucker (and others not mentioned here) drew an erroneous conclusion.

Simply put, metabolism is not locomotion. But, this is a thought for our colleagues in biology to recognize. To confuse locomotion with metabolism is a grave error in Thermodynamics. It is the same as confusing combustion (coal fire) with power to the wheels of a locomotive. It is the same as confusing overeating (obesity) with a way to beat Usain Bolt in the 100 m sprint.

The same reviewer objected that we use the word ‘evolution’ *imprecisely*. He wrote that in animal locomotion “evolution means evolution through natural selection, which includes all the organism’s traits …. survival … success.” How precise is the reviewer’s definition? It is a circular statement (evolution means evolution). Why should nature ‘select’ anything? Why should an organism change, to have ‘survival and ‘success’?

Again, our objective here is not to criticize Darwin, because we have the greatest respect for the few pioneers of his stature. He and his predecessors wrote before thermodynamics existed, that is, before the needed science (physics) and language were available. This is why the doctrine of Darwinism continues to encounter difficulty in the classroom^62^. Again, this is a thought for our colleagues in biology to ponder.

The definition of evolution is in the word itself: *evolvěre*, which means to roll out, forward. The word refers to an object that *changes* in a discernible *direction*. Change and directionality are the essence in the words used by the reviewer (select, survival, success), and in the principle invoked coming from physics (constructal law^1,4,6,13,14,19,22^. Change and directionality are present in all observations of nature, around us, inside of us, and in our hands (the artifacts). Such observations are universal, and this is why evolution is a phenomenon of all physics, which includes animal locomotion, and justifies the biologists’ use of “animal design” as name for their field of study.

In sum, the present article is an invitation to construct ‘the science of form’ and its applications and impact. Intermittency (rhythm) facilitates the evolution of technology^1,62–64^. Vehicles, athletes, and the study of design in nature can benefit from this approach^1,26,65–67^. The wider the range of scales, the greater the opportunity.

Fundamental is the unified view of animal and engineered locomotion. The examples treated in this article have animal and engineered counterparts. Theory is unifying. Theory builds bridges between previously disparate self-standing domains. It is about predicting what is to be found in nature and in the world of artifacts, to continue to empower the individual.

## Methods

Theory, evolutionary design, predictions verified later by comparison with measurements from natural swimmers and fliers.

## Ethical approval

No animal or human was used in the study. No experiments were conducted, therefore experimental protocols were followed. All methods were carried out in accordance with relevant guidelines and regulations.

## Data Availability

None. All the 'data' are in the text of the article.

## Abbreviations

C_D_: Drag coefficient
D: Fuselage diameter, m
D: Length of one scale model, m
D_B_: Body length scale, m
D_1_: Torso length, m
D_2_: Limb length, m
f: Frequency, s^–1^
f: Function, Eqs. (10) and (21), (m/s)^3^
$$\tilde{f}$$: Dimensionless function
F_B_: Body drag force, N
F_1_, F_2_: Forces on two-length model, N
g: Gravitational acceleration, m/s^2^
L: Fuselage length, m
L: Reach, m
L_B_: Body length, m
L_w_: Swept length of wing, m
L_x_: Travel, m, Fig. 1
M: Body mass, kg
S: Wing span, m
t_1_: Push time, s
t_2_: Reach time, s
t_3_: Glide time, s
t_x_, t_y_: Time intervals, s, Fig. 1
t: Wing thickness, m
V: Velocity, m/s
V_B_: Body velocity, m/s
V_1_, V_2_: Velocities during push and reach, m/s
$\tilde{V}_{1}$: Dimensionless velocity, Eq. (35)
W: Work, J
W_x_, W_y_: Work during two-stroke cycle, J, Fig. 1
W_1_: Work done during push, J
W_2_: Work done during reach, J
$$\dot{W}$$: Power, W
X: Ratio, V_2_/V_1_

## Greek symbols

β: Ratio, V_B/_V_1_
ε: Ratio, D_2_/D_1_
η: Ratio, D_1_/D_B_
ρ: Density, body, water, kg/m^3^
ρ_m_: Ambient density, kg/m^3^
